# Islands of spatially discordant APD alternans underlie arrhythmogenesis by promoting electrotonic dyssynchrony in models of fibrotic rat ventricular myocardium

**DOI:** 10.1038/srep24334

**Published:** 2016-04-13

**Authors:** Rupamanjari Majumder, Marc C. Engels, Antoine A. F. de Vries, Alexander V. Panfilov, Daniël A. Pijnappels

**Affiliations:** 1 Laboratory of Experimental Cardiology, Department of Cardiology, Heart Lung Centre Leiden, Leiden University Medical Enter, Leiden, the Netherlands; 2 Department of Physics and Astronomy, Ghent University, Ghent, Belgium

## Abstract

Fibrosis and altered gap junctional coupling are key features of ventricular remodelling and are associated with abnormal electrical impulse generation and propagation. Such abnormalities predispose to reentrant electrical activity in the heart. In the absence of tissue heterogeneity, high-frequency impulse generation can also induce dynamic electrical instabilities leading to reentrant arrhythmias. However, because of the complexity and stochastic nature of such arrhythmias, the combined effects of tissue heterogeneity and dynamical instabilities in these arrhythmias have not been explored in detail. Here, arrhythmogenesis was studied using *in vitro* and *in silico* monolayer models of neonatal rat ventricular tissue with 30% randomly distributed cardiac myofibroblasts and systematically lowered intercellular coupling achieved *in vitro* through graded knockdown of connexin43 expression. Arrhythmia incidence and complexity increased with decreasing intercellular coupling efficiency. This coincided with the onset of a specialized type of spatially discordant action potential duration alternans characterized by island-like areas of opposite alternans phase, which positively correlated with the degree of connexinx43 knockdown and arrhythmia complexity. At higher myofibroblast densities, more of these islands were formed and reentrant arrhythmias were more easily induced. This is the first study exploring the combinatorial effects of myocardial fibrosis and dynamic electrical instabilities on reentrant arrhythmia initiation and complexity.

Remodelling of ventricular tissue is an adaptive response to trauma, disease and ageing. It comprises structural and functional features, including changes in cardiac electrophysiology. Its structural aspects involve changes in cell size, cellular composition and tissue architecture. A key feature of this structural remodelling is cardiac fibrosis, which is characterized by increased numbers and activity of myofibroblasts. Tissue heterogeneity as a consequence of fibrosis, could establish anatomical obstacles creating a substrate for irregular propagation of cardiac action potentials (APs), which promotes wavebreak and thereby predisposes to reentrant arrhythmias^1^,^2^,^3^,^4^. However, wavebreaks can also occur in structurally homogeneous cardiac tissue as a result of dynamically induced functional heterogeneity, such as AP duration (APD) alternans when such heterogeneity is large enough to cause electrotonic load imbalance, a feature promoted by electrical remodelling^5^,^6^,^7^,^8^,^9^. Such imbalance is a well-established source of electrical instabilities^10^.

Electrical communication in cardiac tissue occurs via specialized protein channels called gap junctions, which are concentrated in intercalated discs at the longitudinal ends of cardiomyocytes^8^. Gap junctions are formed when ‘hemichannels’ from neighbouring cardiomyocytes connect. Each hemichannel is composed of an assembly of six polypeptides called connexins. The most common and abundant connexin in the heart is connexin43 (Cx43)^8^,^9^,^10^,^11^,^12^. Cx43 down-regulation and re-localization to the lateral surfaces of cardiomyocytes are prominent features of electrical remodelling in ventricular myocardium^13^,^14^,^15^. Both redistribution of Cx43 and loss of Cx43 expression at the intercalated discs may result in conduction abnormalities like conduction slowing and block, thereby producing a substrate for the development of arrhythmias^16^,^17^,^18^,^19^,^20^,^21^,^22^,^23^.

Early studies have investigated the role of anatomical obstacles in promoting conduction block^24^,^25^ as well as the occurrence of wavebreaks in a homogeneous tissue model with dynamically induced functional heterogeneity in electrophysiological properties^26^. Although the molecular mechanisms underlying arrhythmogenesis in heterogeneous cardiac tissue have been extensively theorized^21^,^24^,^26^,^27^,^28^,^29^,^30^,^31^,^32^,^33^,^34^,^35^, the biophysical consequences of dynamically induced electrotonic imbalances in remodelled cardiac tissue remain poorly understood. One possible mechanism by which such heterogeneity may arise is through APD alternans. APD alternans can either occur as large spatially connected areas of tissue exhibiting consecutive APs of the same phase but with alternating durations (technically referred to as spatially concordant alternans or SCA) or as small connected regions of tissue displaying APs with alternating durations of opposite phase adjacent to one another (technically referred to as spatially discordant alternans or SDA). As SDA promotes spatial dispersion of repolarization^29^,^36^,^37^,^38^,^39^,^40^,^41^, it is mechanistically linked to conduction block and is believed to be more arrhythmogenic than SCA^42^. In combination with tissue heterogeneity arising from mildly elevated levels of myofibroblasts, complex spatiotemporal interactions can be expected to occur prior to arrhythmogenesis. An in-depth biophysical study of these interactions could provide novel mechanistic insights that may help to understand the role of gap junctional remodelling and diffuse fibrosis in creating dynamic electrical instabilities in cardiac tissue.

Therefore, in this paper a head-to-head, synergistic *in silico*-*in vitro* approach was applied for studying the mechanisms underlying arrhythmias in remodelled ventricular tissue, focusing on the effects of Cx43 down-regulation and diffuse cardiac fibrosis. For this purpose, we used (*i*) freshly isolated neonatal rat ventricular cardiomyocytes (NRVMs), and *(ii)* a modified version of the mathematical model of these cells created by Korhonen *et al*.^43^ including the adaptations made by Hou *et al*.^44^. The NRVMs were used to establish confluent monolayers containing ~70% cardiomyocytes and ~30% neonatal rat cardiac myofibroblasts (MFBs) in a random distribution pattern. Intercellular coupling was systemically reduced *in vitro* via RNA interference (RNAi) by incubating the cells with increasing dosages of lentiviral vectors (LVs) encoding Gja1-specific short hairpin (sh) RNAs for selective Cx43 knockdown (Cx43↓) or *in silico* by gradually decreasing the intercellular coupling coefficient. In an earlier study from our group, the ability to inhibit Cx43 expression in cultured MFBs by lentiviral RNAi was proven structurally by immunohistological and western blot analyses and functionally by means of dye transfer assays^45^. In the current study, the same method was used to accomplish Cx43↓ in both NRVMs and MFBs. Programmed electrical stimulation and voltage mapping, together with an interactive data exchange strategy, were used to investigate whether and how these features of ventricular remodelling affected electrical impulse generation and propagation as well as arrhythmia initiation and complexity.

Our results demonstrate for the first time that Cx43↓ and increased myofibroblast density are responsible for a previously unexplored form of complex SDA, characterized by the spatiotemporal evolution of island-like areas of synchronized, oppositely phased APD alternans (designated as alternans phase islands or APIs). Our study not only reveals the presence of APIs in two-dimensional *in silico* and *in vitro* models of ventricular remodelling, but also demonstrates how these local disturbances could lead to the formation of reentrant tachyarrhythmias affecting the whole medium.

## Results

Unless indicated otherwise, figure panels adjacent to a red vertical bar depict *in silico* results, whereas figure panels next to a blue vertical bar represent *in vitro* results.

### Characterization of fibrotic NRVM monolayer cultures

Immunostaining for collagen type I, smooth muscle myosin heavy chain and CD31 (also known as platelet endothelial cell adhesion molecule 1) confirmed that the *in vitro* monolayer cultures consisted of ~70% NRVMs and ~30% MFBs in a random distribution pattern and did not contain vascular smooth muscle cells or endothelial cells (Supplementary Fig. S1). Exposure of these cultures to different dosages of LV. Cx43↓ resulted in a dose-dependent reduction in Cx43 RNA and protein levels (Supplementary Fig. S2), conduction velocity (CV) and wavelength (λ, defined as: APD_80_ × CV; Supplementary Fig. S3). *In vitro* Cx43↓ was accompanied by an increase in reentry inducibility in the fibrotic NRVM cultures (Supplementary Fig. S3).

### High-frequency pacing promotes arrhythmogenesis in fibrotic NRVM monolayer cultures

**Figure 1:**
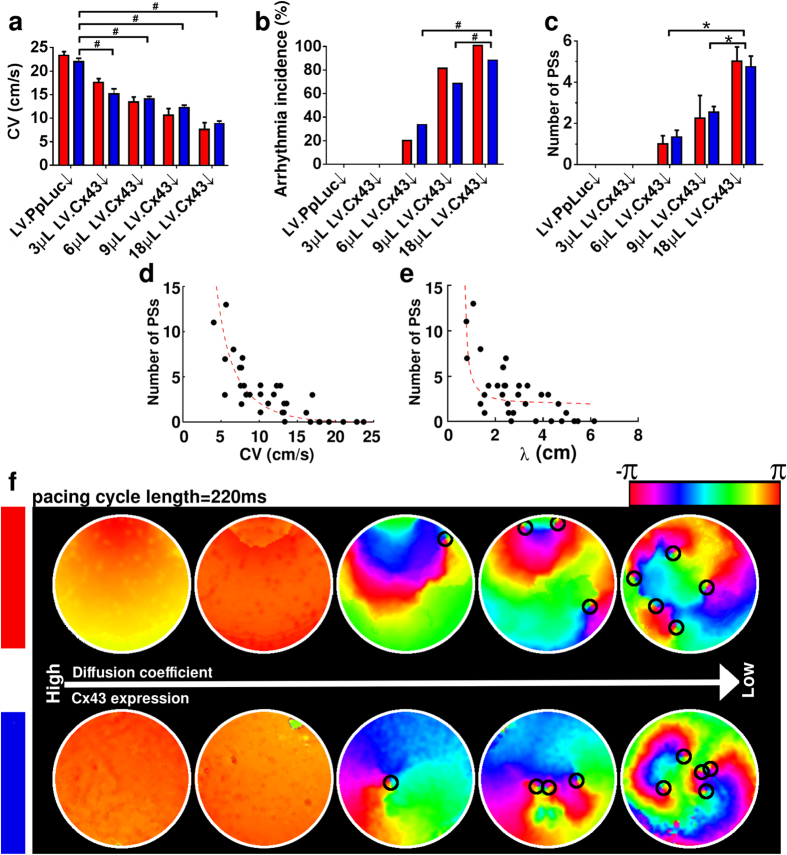
Complexity of electrical activation patterns increases with decreasing CV. Comparison of (**a**) CV, (**b**) arrhythmia incidence and (**c**) arrhythmia complexity (*i.e*., number of PSs) in NRVM monolayers *in silico* (red bars) and *in vitro* (blue bars) at different levels of intercellular coupling and in the presence of 30% interspersed MFBs (N > 9). Statistical analysis was performed by comparing LV.PpLuc↓-transduced cell cultures (negative control) with cell cultures exposed to different amounts of LV.Cx43↓. Statistical significance was expressed as follows: *P < 0.05, ^#^*P* < 0.001. Relationship between arrhythmia complexity and (**d**) CV or (**e**) wavelength (λ), defined as: APD_80_ × CV. (**f**) Pseudocolor plots of phase maps from the *in silico* (top panel) and *in vitro* (bottom panel) datasets. The small black circles indicate the locations of PSs.

High-frequency electrical pacing (3.5 Hz) of our fibrotic NRVM monolayers with the highest degree of Cx43↓ *in vitro* led to complex arrhythmias, *i.e*., reentrant arrhythmias with multiple phase singularities (PSs; points in the phase map where the phase is indeterminate, around which activation wave fronts hinge and progress through a complete cycle from −π to +π; Fig. 1). In order to develop mechanistic insights into the underlying basis of these arrhythmias in a more subtle, precise, controllable and reproducible manner, we employed our *in silico* model. The first step involved validation of this model.

### Validation of the *in silico* model of fibrotic NRVM monolayers

Fibrotic monolayer cultures with 4 different levels of Cx43↓ were studied *in vitro*. Based on the average CVs measured in these monolayers, the *in silico* intercellular coupling constant (see Methods for explanation) was adjusted so that the computer model produced CVs that closely matched the values measured *in vitro* (Fig. 1a). Next, the same pacing protocol was applied *in silico* as *in vitro*. The results from the *in silico* experiments resembled closely those of the *in vitro* studies, demonstrating CV-dependent threshold behaviour for arrhythmia incidence (Fig. 1b) and complexity (Fig. 1c). For both *in silico* and *in vitro* models, stable spiral wave reentry occurred only at CV 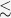 15 cm/s (corresponding to 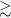 6 μL LV. Cx43↓) and further CV lowering resulted in a similar gradual increase in the number of PSs. Arrhythmia complexity correlated negatively with CV and λ (Fig. 1d,e, respectively). Representative examples of the excitation patterns are shown in Fig. 1f. Thus, we not only developed a minimal *in silico* model for studying the electrophysiological consequences of cardiac remodelling, but also demonstrated its expedience to predict the outcome of *in vitro* experiments.

### Arrhythmogenesis correlates with the development of complex patterns of SDA

**Figure 2:**
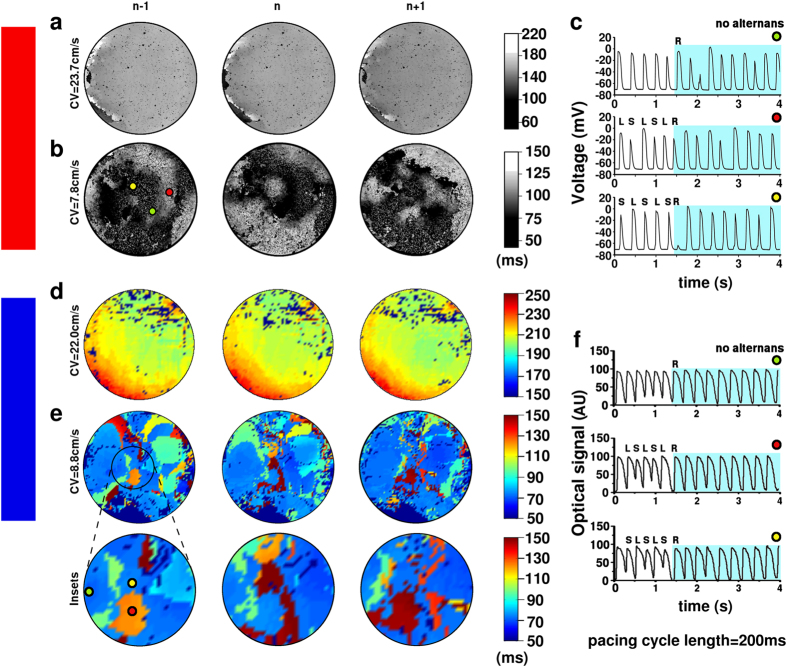
Reentry is preceded by API formation. APD_80_ maps of (**a**,**b**) *in silico* and (**d**,**e**) *in vitro* co-cultures from 3 consecutive beats (n − 1, n and n + 1) at normal (**a**,**d**) and reduced (**b**,**e**) CV. The insets in (**e)** highlight an area showing local alternans phase reversal during successive beats. Time series of (**c**) voltage traces of the *in silico* monolayer shown in (**b**) and (**f**) optical signal traces of the *in vitro* monolayer shown in (**e**) at locations displaying no APD alternans (green dot) or APD alternans of opposite phase beginning either with a LS APD sequence (red dot) or a SL APD sequence (yellow dot). The light blue-colored area in (**c**) and (**f**) show signal recording after the onset of reentry (R). AU, arbitrary units.

Optical mapping recordings of arrhythmogenic *in vitro* cultures showed indications of localized APD alternans just prior to reentry initiation. Similar effects were observed *in silico*. Analysis of *in silico* synchronized APD distribution maps during rapid pacing revealed homogeneous APD distributions in substrates with a high CV (Fig. 2a), as opposed to the development of SDA in the substrates with the lowest CV considered (Fig. 2b). Substrates displaying SDA were characterized by the co-occurrence of three APD patterns: no alternans (NA), alternans with phase long-short (LS) and alternans with phase short-long (SL; Fig. 2c). While the type of SDA reported in previous studies^36^,^37^,^38^,^39^,^46^,^47^ was generally characterized by the development of open nodal lines separating regions of opposite alternans phase, in our model the nodal lines formed closed loops, enclosing regions of a particular alternans phase, which we designated APIs to stress their demarcated nature. To exclude random noise artefacts, we defined APIs as clusters of ≥ 10 connected data points exhibiting alternans of the same phase. APD maps from successive beats demonstrated that areas showing a long APD in beat n − 1, displayed a short APD in beat n and again a long APD in beat n + 1. Areas with a short APD in beat n − 1, showed the inverse behaviour, *i.e*., long and short APDs in beats n and n + 1, respectively. There were also areas where the APD did not change substantially in consecutive beats, indicating absence of APD alternans in these areas. Following the predictions from our *in silico* model, we wrote customized software to generate synchronized APD maps from the optical mapping data generated *in vitro*. In perfect agreement with the *in silico* results, the *in vitro* cultures showed homogeneous synchronized APD maps at normal CV (Fig. 2d), but complex alternans phase distribution patterns at low CVs (Fig. 2e,f).

### Role of APIs in arrhythmogenesis

**Figure 3:**
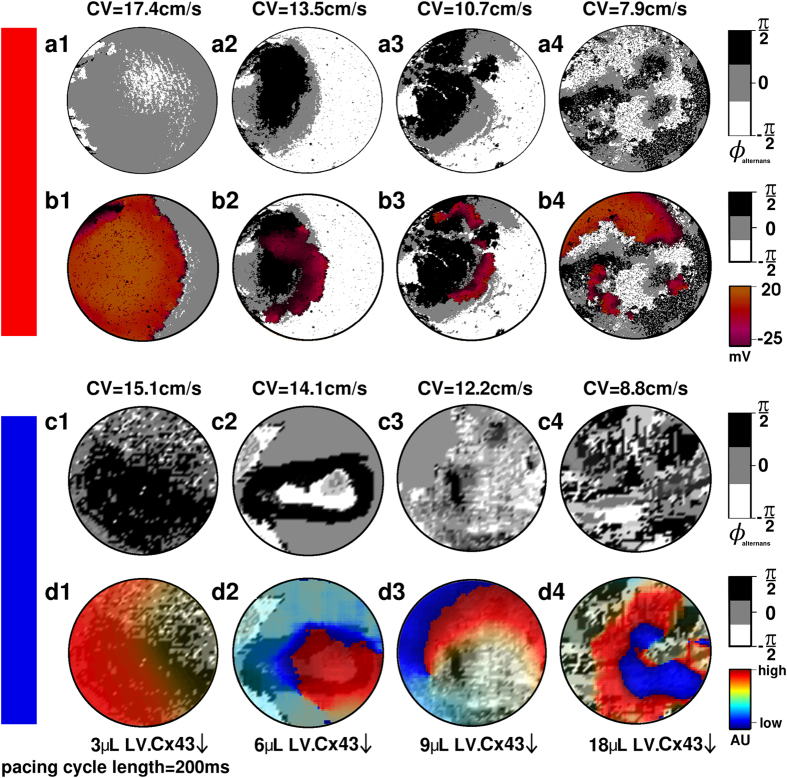
Wavebreaks occur along the borders of APIs. Alternans phase maps of fibrotic (**a**,**b**) *in silico* or (**c**,**d**) *in vitro* NRVM cultures without (**a**,**c**) or with (**b**,**d**) superimposed activation maps just before the onset of reentrant conduction for the monolayers in which reentry could be induced by high-frequency pacing. Formation of APIs and wavebreaks is promoted by CV lowering.

*In silico* analysis revealed that rapid pacing induced wavebreaks along the borders between APIs of opposite alternans phase. This is illustrated at different CVs, by means of alternans phase maps (Fig. 3a1–a4). Superposition of these alternans phase maps with corresponding voltage maps (Fig. 3b1–b4) shows the position(s) of the wavebreak(s). At near normal CV (17.4 cm/s) neither APIs nor wavebreaks occurred. At CV of ~13.5 cm/s, a single large API arose and a wavebreak developed at the border of opposite alternans phase. Further conduction slowing led to higher numbers of (oppositely phased) APIs, thereby increasing wavebreak incidence. A similarly detailed analysis was not possible *in vitro* because of the limited resolution of the imaging setup used for optical voltage mapping. Nonetheless, the formation of APIs *in vitro*, generally also happened at CV < 15 cm/s and their number increased with further Cx43↓ (Fig. 3c1–c4). Moreover, in the fibrotic NRVM cultures wavebreaks always occurred in the vicinity of the borders between adjacent oppositely phased APIs, in consonance with our *in silico* predictions.

**Figure 4:**
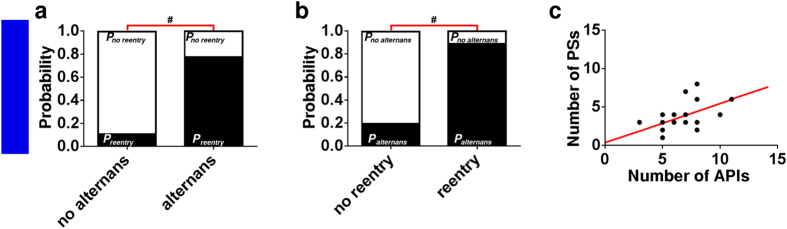
Close linkage between the occurrence of reentry and APD alternans in fibrotic *in vitro* NRVM cultures. (**a**) Probability of successful stable reentry induction in cultures showing APD alternans or no APD alternans. (**b**) Probability of occurrence of APD alternans during high-frequency pacing in cultures showing stable reentry or no reentry induction. (**c**) Relationship between the number of APIs and arrhythmia complexity (*i.e*., the number of PSs).

Time series recordings from different locations in arrhythmic *in vitro* NRVM cultures revealed a significantly higher probability of reentry induction in cultures showing APD alternans than in those without APD alternans (*P* < 0.001; Fig. 4a). Similarly, APD alternans occurred more frequently in cultures with successful stable reentry induction than in cultures that did not display stable reentry following high-frequency pacing (*P* < 0.001; Fig. 4b). Our *in vitro* data furthermore showed a positive correlation between the number of high frequency pacing-induced APIs and reentry complexity (Fig. 4c).

### Origin of APIs

**Figure 5:**
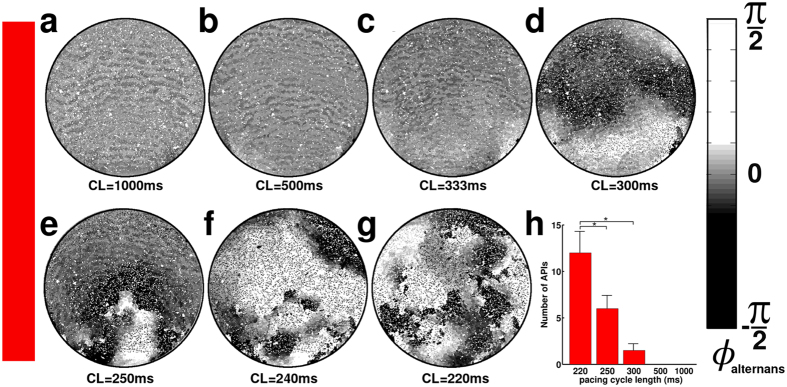
High-frequency pacing plays an essential role in API formation. Alternans phase maps of fibrotic *in silico* NRVM cultures corresponding to the experimental group with the lowest CV, paced at different cycle lengths (CLs) reveal a direct correlation between pacing frequency and API incidence.

**Figure 6:**
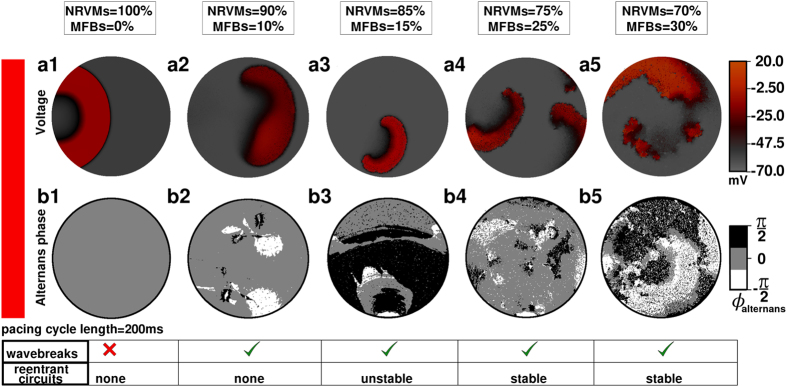
Role of MFBs in reentry initiation: insights from the *in silico* model. **(a**) Voltage and (**b**) alternans phase maps of *in silico* NRVM cultures corresponding to the experimental group with the lowest CV, containing different percentages of randomly distributed MFBs. Alternans was not observed in monolayers with 100% cardiomyocytes (**a1**,**b1**); in the presence of 10% MFBs, some portions of the monolayer began to show unstable alternans (**a2**,**b2**). In this scenario wavebreaks could occur, but these failed to develop into reentrant circuits. With 15% MFBs (**a3**,**b3**) a limited number of large APIs began to form and; unstable reentry could be initiated. With 25% MFBs (**a4**,**b4**), multiple APIs started to form, which evolved dynamically giving rise to stable reentrant circuits. Raising the percentage of MFBs to 30% further increased the pro-arrhythmicity of the substrate (**a5**,**b5**).

Having made the association between reentry initiation, the complexity of reentry patterns and API formation, the origin of these APIs was investigated next. Specifically, the combinatorial effects of structural discontinuities (MFBs) and functional instabilities (high frequency pacing-induced alternans) on the onset of reentry were studied *in silico*. As shown in Fig. 5a–h, in monolayers with 30% randomly distributed MFBs, APIs only occurred at cycle lengths <333 ms and their number increased with a further decrease in cycle length. For investigations into the role of structural discontinuities in API formation, computer simulations of NRVM cultures containing different percentages of MFBs and a CV of ~7.9 cm/s (*i.e*., the lowest CV analysed) were performed. High-frequency paced NRVM cultures without MFBs showed that APD alternans was practically absent (Fig. 6a1,b1) and wavebreaks were not observed. In monolayers with 10% randomly distributed MFBs, APD alternans began to appear (Fig. 6a2,b2). Large APIs emerged when ~15% of the cells were MFBs (Fig. 6a3,b3). Stable API-mediated reentry was observed in monolayers containing ≥ 25% MFBs (Fig. 6a4,b4,a5,b5), and at > 40% MFBs conduction block occurred. Thus, the presence of interspersed MFBs above a certain critical percentage could be recognized as a factor involved in the formation of APIs. Taken together, our data indicate the co-occurrence of (*i*) tissue heterogeneity (*e.g*., diffuse fibrosis) and (*ii*) CV slowing below a certain threshold (*e.g*., by Cx43 down-regulation) is required for successful API formation upon high-frequency pacing. Furthermore, increased numbers of MFBs lowers the threshold of CV reduction and decreased CV lowers the threshold of fibrosis necessary for API formation.

## Discussion

The key findings of this study are: (*i*) Rapid electrical pacing of fibrotic NRVM cultures showed a Cx43 expression-dependent threshold behaviour towards the development of stable reentrant arrhythmias in a model of ventricular remodelling-associated arrhythmogenesis; (*ii*) Arrhythmia complexity increased with decreasing Cx43 expression; (*iii*) The occurrence of complex arrhythmias was attributed to a decrease in electrotonic synchronization linked to the presence of MFBs; (*iv*) The increased dyssynchrony manifested itself as a special type of spatially discordant APD alternans characterized by APIs; (*v*) Reentrant arrhythmia incidence and complexity positively correlated with the number of APIs.

Ventricular remodelling is a dynamic process of alterations in size, shape and function of the ventricles in response to cardiac injury (*e.g*., myocardial infarction) and stress (*e.g*., pressure or volume overload). The contributing cellular events include cardiomyocyte death, cardiomyocyte hypertrophy, hyperplasia of cardiac fibroblasts (CFBs), CFB-MFB transformation, excessive extracellular matrix deposition and electrical remodelling^48^,^49^. These events cause structural and functional disruptions of the cardiac syncytium, which contribute to a loss of force-generating capacity and the development of cardiac arrhythmias.

Several studies have shown that ventricular remodelling predisposes to the development of APD alternans following high-frequency pacing^50^,^51^. NRVM monolayers treated with Bay K8644^46^ displayed high-frequency pacing-induced SDA, characterized by the co-occurrence of APD and [Ca^2+^]_i_ alternans with detectable nodal lines separating regions that alternated out of phase, suggesting that SDA is a dynamically generated phenomenon, predisposing to arrhythmias. A common aspect of electrical remodelling is Cx43 down-regulation and lateralization, which is associated with conduction slowing and arrhythmogenesis. Suppression of SDA in Langendorff-perfused guinea pig hearts by rotigaptide, which enhances gap junctional communication, suggests that intercellular uncoupling indeed plays an important role in the development of SDA^47^,^52^.

Ventricular remodelling includes both structural and electrical changes, the independent consequences of which have been discussed above. However, the biophysical consequences of complex interactions between structural discontinuities and dynamically induced functional instabilities, and in particular their functional interdependencies (*e.g*., the influence on electrotonic balance, electrical signal propagation and synchronization as well as correlation with reentry complexity) remains poorly understood. This study aimed at addressing the role of these complex interactions in arrhythmogenesis using an interactive *in silico-in vitro* approach.

Our *in silico* monolayer studies revealed the existence of a direct relationship between the number of high frequency pacing-induced APIs and the percentage of interspersed MFBs (Fig. 6). This association is in line with findings by Woo *et al*.^53^, Kizana *et al*.^54^ and Engelman *et al*.^55^ In more detail, Woo *et al*.^53^ showed that in NRVM monolayers, spatial heterogeneities related to the presence of CFBs can cause some nontrivial wave dynamics leading to complex reentrant conduction patterns. Kizana *et al*.^54^ demonstrated that CFBs can modulate the excitability of cardiomyocytes in a Cx43-dependent manner. They explored the effect of Cx43-negative mouse fibroblasts on the intrinsic beat frequency of NRVMs cultured on top. NRVMs on top of wild-type fibroblasts (with native Cx43 levels) exhibited a significantly lower beating rate compared to those grown on fibroblasts lacking functional Cx43. Forced expression of Cx43 in fibroblasts from Cx43 knockout mice led to a near normalization of beating frequency, demonstrating that fibroblasts play an important role in modulating the excitability of NRVMs through gap junctional coupling. Furthermore, in an *in silico* study, Engelman *et al*.^55^ found that SDA occurs at lower pacing frequencies and more often in fibrotic than in non-fibrotic cardiac tissue as a result of discontinuous conduction through the disrupted cardiac syncytium. Such abnormal AP propagation causes large local fluctuations in the diastolic intervals giving rise to regional electrotonic instability. These instabilities modulate the CV spatiotemporally and influence APD restitution.

We hypothesize that when such instabilities occur in close proximity of each other, the electrotonic balance of the system is perturbed. The propagating wavefront becomes fractionated, while island-like zones emerge, exhibiting synchronized electrophysiological behaviour (APD alternans of common phase). In this scenario, the size of an API is determined by the length scale of the influence of the localized instabilities. If the instabilities emerge distant from each other their mutual influence is small and an API may not form. However, if a region is highly fibrotic, it will sustain many synchronized instabilities and show a high propensity for API development. The dynamic state of the tissue prior to pacing^39^,^56^,^57^, 39,56-57 and short-term memory^37^ are also likely to influence the development of APIs and their spatiotemporal distributions. Although previous studies mostly reported the occurrence of SDA characterized by open nodal lines, there are some studies that demonstrate island-like SDA patterns in explanted whole hearts^38^,^57^. However, none of these studies, explored their relevance, origin or contribution to the development of arrhythmogenesis. Our work differs from earlier reports in that we made use of heterogeneous tissue models incorporating diffuse fibrosis. We found that diffuse fibrosis and Cx43 knockdown synergistically reduced the excitability of cardiac tissue, thereby causing fractionation of the propagating wavefront. This fractionation enhances localized electrical resynchronization leading to APIs. However, the exact mechanism by which such resynchronization results in APIs of specific shape is not completely clear and requires special investigation that lies beyond the scope of this study. Additional research into the genesis and dynamics of APIs, including the relative abundance and distribution of each APD pattern and the role of short-term memory in API development is warranted. Furthermore, in might be of interest to investigate [Ca^2+^]_i_ dynamics during API formation in future studies.

**Figure 7:**
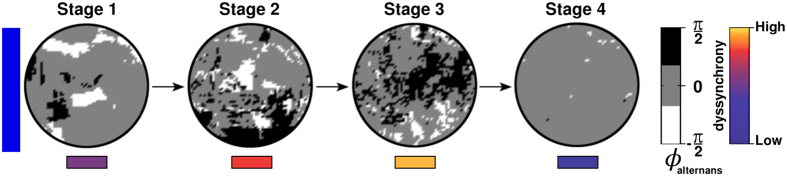
Conduction slowing causes progressive dyssynchrony leading to reentry initiation. In substrates with reduced CV, reentry initiation can be divided in 4 stages. In the first stage, high-frequency pacing induces patchy APD alternans leading to the formation of APIs and the substrate first begins to develop functional dyssynchrony. In the second stage, under the influence of electrotonic effects, these APIs evolve dynamically, drifting towards each other, such that APIs of opposite phases align side-by-side. The substrate thus develops SDA and dyssynchrony is increased. In the third phase, electrical interactions between adjacent APIs forces them to break up into smaller, disconnected API, thereby maximizing dyssynchrony within the substrate. This leads to wavebreaks, which, in the fourth and final stage of the process, mature into stable reentrant circuits in which spatial APD alternans disappears.

As shown in Fig. 3, a decrease in CV was associated with voltage alternans. The kind of alternans (spatially concordant or discordant) depended on the level of intercellular uncoupling. In NRVM cultures with modest Cx43↓, large connected areas without APD alternans co-existed with one or few large APIs. In cultures with a high degree of intercellular uncoupling, pacing above a critical frequency led to the development of more complex SDA patterns, characterized by multiple APIs of different size and phase. These APIs also appeared in the *in silico* model (Fig. 2). Once APIs are formed, the substrate is prone to develop wavebreaks. These wavebreaks formed along the boundaries between adjacent APIs of opposite phase (Fig. 3). At normal CVs the formation of APIs was inhibited. If the excitation wavelength is such that at any instant the substrate has enough recovered area to support one stable reentrant circuit, only the best positioned wavebreak gives rise to a sustained spiral wave. The optimal position seems to be determined by the degree of source-sink mismatch encountered by the wavebreak. Conduction slowing was associated with increased electrical dyssynchrony, exemplified by the formation of multiple APIs of opposite phase. During high-frequency pacing, APIs evolved dynamically, drifting towards one another until they appeared side-by-side. At maximal dyssynchrony (Fig. 7, stage 3), electrotonic interaction between adjacent APIs of opposite phase resulted in pacing-induced wavebreaks at the islands’ borders. Higher dyssynchrony prior to reentry initiation correlated with more PSs.

Our data reveal that at higher MFB percentages a smaller reduction in Cx43 level suffices to induce APIs: with 30% MFBs, API induction is initiated at a CV of ~13.5 cm/s, while for 10% of MFBs APIs started to occur at a CV of ~7.9 cm/s (Fig. 3). Similarly, the level of fibrosis required for reentrant circuit formation is directly proportional to the Cx43 level. As seen from Fig. 6, at a CV of ~7.9 cm/s, stable API-mediated reentry requires at least 25% MFBs, whereas a monolayer with a CV of ~13.5 cm/s requires up to 30% MFBs for stable arrhythmias to occur. A monolayer with a CV as high as ~17.4 cm/s cannot support APIs even at 30% MFBs.

Electrotonic effects sometimes caused small APIs to merge, thereby decreasing the number of possible wavebreak initiation points. This could explain why in some cases although the substrate initially supported multiple spiral waves their number decreased over time with fewer stable reentrant circuits remaining. Stabilization of reentrant circuits via the onset of stable reentry resulted in disappearance of asynchronous APD alternans (Fig. 7). In our *in vitro* studies < 1% of the co-cultures were excluded because they showed spontaneous activity. As cardiomyocyte-fibroblast interaction can also result in the onset of oscillatory dynamics^58^,^59^,^60^, it would be interesting to study the origin of arrhythmias in such co-cultures and their relation to fibrosis, as a future project.

In extended systems (*i.e*., cardiac monolayers, tissue preparations and whole hearts), alternans has been found to occur either synchronously (SCA) over large connected areas or asynchronously (SDA) over smaller regions separated from each other through nodal lines/surfaces. Although there is extensive literature attempting to correlate the dynamics of nodal lines to the underlying alternans mechanism^38^,^39^,^40^,^56^,^57^,^61^,^62^,^63^, these studies mostly concerned the behaviour of nodal lines arising from SDA induced by CV restitution or tissue heterogeneity. In this study, we have been able to associate this unusual type of alternans with a pathological substrate and to identify a mechanism by which these APIs could increase pro-arrhythmic risk. This may provide relevance to our study from a clinical perspective.

## Materials and Methods

Detailed technical information can be found in the Supplementary Information.

### Numerical methods

The temporal electrophysiological behaviour of a single NRVM was described by using an ordinary differential equation:


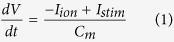


where *V* is the transmembrane voltage in mV, t is time in ms, *C*_*m*_ is the capacitance per unit surface area of the cell in μF/cm^2^, *I*_*stim*_ is the external current stimulus, and *I*_*ion*_ is the total ionic current flowing across the cell membrane. All currents are expressed in pA/pF. I_ion_ represents the sum of 12 major and minor ionic currents:


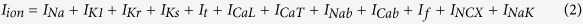


where *I*_*Na*_ is the fast Na^+^ current, *I*_*K1*_ is the time-independent K^+^ current, *I*_*Kr*_ is the rapid delayed rectifier K^+^ current, *I*_*Ks*_ is the slow delayed rectifier K^+^ current, *I*_*t*_ is the transient outward K^+^ current, *I*_*CaL*_ is the L-type Ca^2+^ current, *I*_*CaT*_ is the T-type Ca^2+^ current, *I*_*Nab*_ and *I*_*Cab*_ are the background Na^+^ and Ca^2+^ currents, respectively, *I*_*f*_ is the hyperpolarization-activated current, *I*_*NCX*_ is the Na^+^/Ca^2+^ exchanger current, and *I*_*NaK*_ is the Na^+^/K^+^ ATPase current. The flow of currents in and out of the cells is controlled by ion channels, which were modelled as conductances. The opening and closing of these ion channels are controlled by gates with specific time constants. The formulation of these currents, as well as the model parameters and constants are listed in Hou *et al*.^44^ Our new formulation of the Ca^2+^ dynamics of the cell is presented in the Supplementary Information.

The transmembrane potential of the NRVMs in the monolayer studies was governed by the following reaction-diffusion equation:


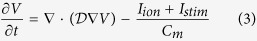


where 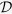 is the symmetric tensor whose elements determine the degree of electrical conductance in each direction of propagation. In order to maintain consistency with our *in vitro* set up, anisotropy was disregarded. This reduced 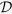 to a scalar coupling constant *D* multiplied by an identity matrix^64^. Thus in our monolayer simulations:


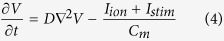


This equation was subject to Neumann zero-flux boundary conditions. The numerical details are provided in the Supplementary Information.

Thirty percent randomly distributed MFBs were introduced in the simulation domain. The MFBs were modelled using the passive formulation of MacCannell *et al*.^65^ A gap junctional coupling coefficient (G_gap_) of 0.5 nS/pF was used for intercellular coupling between NRVMs and MFBs^66^.

The possible connections between NRVMs and MFBs in the *in silico* model are shown in Supplementary Fig. S4a. Details of the MFB model and the model for natural cellular heterogeneity are also provided in the Supplementary Information. The AP characteristics were computed from a single mathematical cell and validated with existing literature (Supplementary Fig. S4b,c). The APD restitution curve derived from our *in silico* model lies in between those based on the *in vitro* and *in silico* data published by Hou *et al*.^44^ (Supplementary Fig. S4d).

### *In silico* pacing protocol

The *in silico* monolayer was paced initially at 1000-ms cycle length for 3 s, with an electrical pulse strength of 100 pA and a duration of 2 ms. Next, the cycle length was gradually decreased in steps of 150 ms, taking care to pass 3–4 pulses at each cycle length until 1:1 capture was no longer observed or arrhythmia was initiated.

### Experimental methods

All animal experiments were approved by the Animal Experiments Committee of Leiden University Medical Centre (LUMC) and conformed to the Guide for the Care and Use of Laboratory Animals as stated by the United States National Institutes of Health.

### Cell isolation and culture

NRVMs and cardiac MFBs were isolated and cultured as described previously^67^. Briefly, hearts were excised from anaesthetized neonatal rats, and ventricular tissue was finely minced and dissociated with collagenase type 1 (450 U/mL; Worthington, Lakewood, NJ) and DNase I (18.75 Kunitz/mL; Sigma-Aldrich, St. Louis, MO). Our cell isolation protocol allowed us to obtain NRVMs with a baseline 15–20% contamination of MFBs. Therefore, after two consecutive pre-plating steps, the purified NRVMs were mixed with 10–15% neonatal rat MFBs, such that the final co-cultures had a ratio of 70 NRVMs:30 MFBs. The cells were seeded on fibronectin (Sigma-Aldrich)-coated glass coverslips in 24-well cell culture plates (Corning Life Sciences, Corning, NY). Cells were plated at a total density of 1–7 × 10^5^ cells/well, depending on the assay, and treated for 2 hours with mitomycin-C (10 μg/mL; Sigma-Aldrich) to prevent proliferation of non-cardiomyocytes^67^.

### RNAi

Cx43 expression in NRVMs was selectively and dose-dependently inhibited using self-inactivating LV particles encoding two different rat Gja1 gene-specific shRNAs. The shuttle constructs used to generate the LVs are derivatives of plasmid clones TRCN0000348381 and TRCN0000068474 from the MISSION shRNA library (Sigma-Aldrich) in which the marker gene cassette consisting of the human phosphoglycerate kinase 1 gene promoter, the *Streptomyces alboniger* puromycin-N-acetyltransferase-coding sequence and, in case of TRCN0000348381, the woodchuck hepatitis virus posttranscriptional regulatory element was substituted with the human eukaryotic translation elongation factor 1 alpha 1 gene promoter and the *Aequorea victoria* enhanced green fluorescent protein-coding sequence. The resulting LVs were designated LV. Cx43↓ or LV. Cx43_1_↓ and LV. Cx43_2_↓, respectively. The negative control vector (LV.PpLuc↓) had the same genetic makeup, except that it contained the *Photinus pyralis* luciferase (PpLuc)-specific shRNA-coding sequence of plasmid SHC007 (Sigma-Aldrich) instead of a rat Gja1-specific shRNA-coding sequence.

### Optical voltage mapping

On day 9 of culture, assessment of AP propagation in cellular monolayers was performed by optical mapping using di-4-ANEPPS (Life Technologies) as potentiometric dye, as described previously^67^. Optical mapping was carried out with a MiCAM ULTIMA-L imaging system (SciMedia USA, Costa Mesa, CA). Optical signals were recorded at a 6-ms frame rate and analysed using BrainVision Analyzer 13.12.20 software (Brainvision, Tokyo, Japan). Based on the outcome of our *in silico* studies, the cultured monolayers were stimulated by electrical pacing with an epoxy-coated bipolar platinum electrode with square supra-threshold electrical stimuli, at a frequency predicted to induce arrhythmias.

### Alternans phase maps

To construct alternans phase maps, we considered APD maps from 3 successive beats, designated n − 1, n, and n + 1. The alternans phase (*ϕ*_*alternans*_) at any point within the monolayer was calculated as:


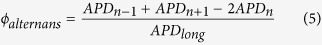



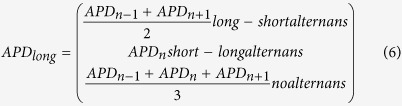


*ϕ*_*alternans*_ was then binned into 3 groups, with labels π/2 (*ϕ*_*alternans*_ ≥ 0.6), 0 (−0.6 < *ϕ*_*alternans*_ < 0.6) and −π/2 (*ϕ*_*alternans*_ ≤ −0.6), to signify long-short, non-alternating and short-long APD sequences, respectively.

### Statistics

Statistical analyses were performed using GraphPad Prism software version 6.02 (GraphPad Software, La Jolla, CA). Unpaired Student’s t test and Fisher’s exact test were used for comparisons between experimental groups, as appropriate. Data were expressed as mean ± standard error of mean for a specified number (N) of observations. Results were considered statistically significant at *P* values < 0.05. Statistical significance was expressed as follows: *P < 0.05, ^#^*P* < 0.001 or NS: not significant. Non-linear regression curves were constructed by using robust exponential or hyperbolic 1-phase decay curve fits. Accuracy was expressed as coefficient of determination (R^2^). Arrythmia complexity was defined as the number of PSs per monolayer culture (surface area 1.8 cm^2^). Phase maps were constructed with dedicated software using the phase space method, as described previously^68^.

## Additional Information

**How to cite this article**: Majumder, R. *et al*. Islands of spatially discordant APD alternans underlie arrhythmogenesis by promoting electrotonic dyssynchrony in models of fibrotic rat ventricular myocardium. *Sci. Rep*. **6**, 24334; doi: 10.1038/srep24334 (2016).

## Supplementary Material

Supplementary Information
