# Supplementary material for: Islands of spatially discordant APD alternans underlie arrhythmogenesis by promoting electrotonic dyssynchrony in models of fibrotic rat ventricular myocardium
Source: Sci Rep. 2016 Apr 13;6:24334. doi: 10.1038/srep24334 (PMC4829862; doi:10.1038/srep24334)
Supplement: Supplementary Information [file srep24334-s1.doc]

**Islands of spatially discordant APD alternans underlie arrhythmogenesis by promoting electrotonic dyssynchrony in models of fibrotic rat ventricular myocardium.**

*Majumder & Engels, et al.: Phase islands and arrhythmogenesis*

Rupamanjari Majumder, PhD^1,†^; Marc C. Engels, MD^1,†^; Antoine A.F. de Vries, PhD^1†^; Alexander V. Panfilov, PhD^2†^*, Daniël A. Pijnappels, PhD^1†^*

^†^ These authors contributed equally to this work.

^1^ Laboratory of Experimental Cardiology, Department of Cardiology, Heart Lung Centre Leiden, Leiden University Medical Enter, Leiden, the Netherlands.

^2^ Department of Physics and Astronomy, Ghent University, Ghent, Belgium.

*Correspondence to: Daniël A. Pijnappels, PhD, Laboratory of Experimental Cardiology, Department of Cardiology, Leiden University Medical Center, 2300 RC Leiden, the Netherlands.

E-mail: [D.A.Pijnappels@lumc.nl](mailto:D.A.Pijnappels@lumc.nl) or Alexander V. Panfilov PhD, Department of Physics and Astronomy, Ghent University, Ghent, Belgium. E-mail: [alexander.panfilov@ugent.be](mailto:alexander.panfilov@ugent.be)

**SUPPLEMENTAL MATERIAL**

**Numerical Methods**

In our cardiomyocyte model, the Ca^2+^ core structure characteristic of the models by Korhonen *et al.*^1^ and Hou *et al*.,^2^ was replaced by a single chamber with a single Ca^2+^ component ([Ca]_i_) that accounts for the cytosolic Ca^2+^, the subsarcolemmal Ca^2+^, the Ca^2+^ of the subsarcoplasmic reticulum and the Ca^2+^ in between. For the study presented in this paper, cell contraction via excitation is neither a feature of interest, nor does it have, as we have checked, any significant influence on the qualitative results. Thus, this modification enabled us to increase the computational speed of the model while preserving the essential electrophysiological behavior of its predecessors.

**Ca^2+^ related equations:**

***L-type* Ca^2+^ current:**

$$\begin{matrix} I_{\text{CaL}}=4G_{\text{CaL}}\text{dff}_{\text{Ca}}V\frac{F^{2}}{\text{RT}}\frac{\left(\text{Ca}_{i}e^{\left(\frac{\text{2VF}}{\text{RT}}\right)}-\text{0.341Ca}_{o}\right)}{e^{\left(\frac{\text{2VF}}{\text{RT}}\right)}-1.0},\left(1\right) \\ d_{\infty}=\frac{1.0}{\text{1.0+e}^{\frac{\left(-11.1-V\right)}{7.2}}},\left(2\right) \\ \alpha_{d}=0.25+\frac{1.4}{\text{1+e}^{\left(\frac{-\left(\text{35+V}\right)}{13}\right)}},\left(3\right) \\ \beta_{d}=\frac{1.4}{\text{1+e}^{\left(\frac{\left(\text{V+5.0}\right)}{5.0}\right)}},\left(4\right) \\ \gamma_{d}=\frac{1.0}{\text{1+e}^{\left(\frac{\left(50.0-V\right)}{20}\right)}},\left(5\right) \\ \tau_{d}\text{=α}_{d}\beta_{d}\text{+γ}_{d},\left(6\right) \end{matrix}$$

$$\begin{matrix} \frac{\text{dd}}{\text{dt}}=\frac{d_{\infty}-d}{\tau_{d}},\left(7\right) \\ f_{\infty}=\frac{1.0}{\text{1.0+e}^{\left(\frac{\left(\text{23.3+V}\right)}{5.4}\right)}},\left(8\right) \\ \tau_{f}=1125e^{\left(\frac{-\left(\text{V+27.0}\right)^{2}}{240}\right)}+\frac{165}{\text{1+e}^{\left(\frac{\left(25-V\right)}{10}\right)}}+\text{120,}\left(9\right) \\ \frac{\text{df}}{\text{dt}}=\frac{f_{\infty}-f}{\tau_{f}},\left(10\right) \\ \alpha_{\text{fCa}}=\frac{1.0}{1+\left(\frac{\text{Ca}_{i}}{0.325}\right)^{8}},\left(11\right) \\ \beta_{\text{fCa}}=\frac{0.1}{\text{1+e}^{\left(\frac{\text{Ca}_{i}-0.5}{0.1}\right)}},\left(12\right) \\ \gamma_{\text{fCa}}=\frac{0.2}{\text{1+e}^{\left(\frac{\text{Ca}_{i}-0.75}{0.8}\right)}},\left(13\right) \\ f_{\text{Ca}\infty}=\frac{\alpha_{\text{fCa}}\text{+β}_{\text{fCa}}\text{+γ}_{\text{fCa}}+0.23}{1.46},\left(14\right) \\ \tau_{\text{fCa}}=\text{10ms,}\left(15\right) \\ \frac{\text{df}_{\text{Ca}}}{\text{dt}}\text{=k}\frac{f_{\text{Ca}\infty}-f_{\text{Ca}}}{\tau_{\text{fCa}}},\left(16\right) \end{matrix}$$

where, $\text{k=}\left\{\begin{matrix} 0f_{\text{Ca}\infty}\text{>f}_{\text{Ca}}\text{,V>}-\text{60mV} \\ \text{1otherwise} \end{matrix}\right\}\left(17\right)$

**Ca^2+^ fluxes:**

$$\begin{matrix} J_{\text{rel}}\text{=ν}_{1}P_{o1}\left(\text{Ca}_{\text{JSR}}-\text{Ca}_{i}\right),\left(18\right) \\ J_{\text{leak}}\text{=k}_{\text{leak}}\left(\text{Ca}_{\text{NSR}}-\text{Ca}_{i}\right),\left(19\right) \\ J_{\text{tr}}=\frac{\left(\text{Ca}_{\text{NSR}}-\text{Ca}_{\text{JSR}}\right)}{\tau_{\text{tr}}},\left(20\right) \end{matrix}$$

**Ca^2+^ buffering:**

$$\beta_{\text{SR}}=\frac{1.0}{1.0+\frac{\left[\text{CSQN}\right]_{\text{tot}}K_{\text{mCSQN}}}{\left(\text{Ca}_{\text{JSR}}\text{+K}_{\text{mCSQN}}\right)^{2}}},\left(21\right)$$

**Ryanodine receptor gating:**

$$\begin{matrix} K_{\text{mRyR}}=\frac{3.51}{\text{1.0+e}^{\left(\frac{\left(\text{Ca}_{\text{JSR}}-530.0\right)}{200.0}\right)}}+\text{0.25,}\left(22\right) \\ P_{C1}=1-P_{o1},\left(23\right) \\ \frac{\text{dP}_{\text{o1}}}{\text{dt}}\text{=k⁺}\frac{\text{Ca}_{i^{N}}}{\text{Ca}_{i^{N}}\text{+K}_{\text{mRyR}^{N}}}P_{C1}-\text{k⁻P}_{\text{o1}},\left(24\right) \end{matrix}$$

**Sarcoplasmic/Endoplasmic Reticulum Ca^2+^ ATPase (SERCA):**

$$\begin{matrix} J_{\text{up}}=\frac{V_{\text{maxf}}\left(\frac{\text{Ca}_{i}}{K_{\text{mf}}}\right)^{H_{f}}-V_{\text{maxr}}\left(\frac{\text{Ca}_{\text{NSR}}}{K_{\text{mr}}}\right)^{H_{r}}}{1.0+\left(\frac{\text{Ca}_{i}}{K_{\text{mf}}}\right)^{H_{f}}+\left(\frac{\text{Ca}_{\text{NSR}}}{K_{\text{mr}}}\right)^{H_{r}}},\left(25\right) \\ J_{\text{CaSR}}\text{=J}_{\text{rel}}-J_{\text{up}}\text{+J}_{\text{leak}},\left(26\right) \\ J_{\text{CaSL}}=\left(2I_{\text{NCX}}-I_{\text{CaL}}-I_{\text{CaT}}-I_{\text{Cab}}\right)\frac{A_{\text{cap}}C_{m}}{2F\times10^{-6}},\left(27\right) \end{matrix}$$

**Ca^2+^ release and uptake:**

$$\begin{matrix} \frac{\text{dCa}_{\text{NSR}}}{\text{dt}}=\frac{\left(J_{\text{up}}-J_{\text{leak}}-J_{\text{tr}}\right)}{V_{\text{NSR}}},\left(28\right) \\ \frac{\text{dCa}_{\text{JSR}}}{\text{dt}}\text{=β}_{\text{SR}}\frac{\left(-J_{\text{rel}}\text{+J}_{\text{tr}}\right)}{V_{\text{JSR}}},\left(29\right) \\ \beta_{\text{Cai}}=\frac{1.0}{1+\frac{\left[\text{TRPN}\right]_{\text{tot}}K_{\text{mTRPN}}}{\left(\text{Ca}_{i}\text{+K}_{\text{mTRPN}}\right)^{2}}+\frac{\left[\text{CMDN}\right]_{\text{tot}}K_{\text{mCMDN}}}{\left(\text{Ca}_{i}\text{+K}_{\text{mCMDN}}\right)^{2}}},\left(30\right) \\ \frac{\text{dCa}_{i}}{\text{dt}}\text{=β}_{\text{Cai}}\frac{\left(J_{\text{CaSR}}\text{+J}_{\text{CaSL}}\right)}{V_{\text{myo}}},\left(31\right) \end{matrix}$$

All concentrations were measured in micromolar ($\text{μM}$) and all time constants in milliseconds (ms).

**Simulation Details:**

A simulation domain containing $576\times576$ grid points was used with spatial resolution

$\text{δx}$ = 0.003125 cm and time step $\text{δt}$ = 0.002 ms, 0.005 ms, 0.01 ms or 0.02 ms depending on the conduction velocities (CVs). The simulations were performed on a circular domain, with a diameter of 1.56 cm*,* in keeping with the diameter of a well from a 24-well cell culture plate*.*

**Modeling Myofibroblasts (MFBs):**

The formulation for the myocyte-MFB coupling was as follows:

$$\begin{matrix} \frac{\partial V_{m}}{\partial t}=\frac{-I_{\text{ion}}}{C_{m}}\text{+Diff}_{m},\left(32\right), \\ \frac{\partial V_{f}}{\partial t}\text{=Diff}_{f}\left(33\right), \end{matrix}$$

where *V_m_* is the transmembrane voltage across myocytes, *V_f_* is the transmembrane voltage across MFBs, *Diff_m_* is the diffusion term for myocytes and *Diff_f_* is the diffusion term for MFBs. The resting membrane potential of the MFBs was chosen to be *-*20 mV. The diffusion term for myocytes was computed (see Fig. S1a) as $\text{Diff}_{m}=\sum_{\text{n=1}}^{4} a_{n}.$ Here $a_{n}$ is the term for electrical coupling to the *n^th^* neighboring celle; (*n=1* for the neighbor *(i-1,j)*, *n=2* for the neighbor *(i+1,j)*, *n=3* for the neighbor *(i,j-1)*, and *n=4* for the neighbor *(i,j+1)*. Table I lists the values of $a_{n}$ when the central cell was a myocyte and the *n^th^* neighboring cell was a myocyte or an MFB.

 Table I: Computing the diffusion term for Eq.32

| $a_{n}$ | neighbor: NRVM | neighbor: MFB |
| --- | --- | --- |
| $a_{1}$ | $\frac{D_{\text{mm}}}{\left(\text{δx}\right)^{2}}\left(V_{m}\left[i-1\right]\left[j\right]-V_{m}\left[i\right]\left[j\right]\right)$ | $\frac{G_{\text{gap}}C_{f}}{C_{m}}\left(V_{f}\left[i-1\right]\left[j\right]-V_{m}\left[i\right]\left[j\right]\right)$ |
| $a_{2}$ | $\frac{D_{\text{mm}}}{\left(\text{δx}\right)^{2}}\left(V_{m}\left[\text{i+1}\right]\left[j\right]-V_{m}\left[i\right]\left[j\right]\right)$ | $\frac{G_{\text{gap}}C_{f}}{C_{m}}\left(V_{f}\left[\text{i+1}\right]\left[j\right]-V_{m}\left[i\right]\left[j\right]\right)$ |
| $a_{3}$ | $\frac{D_{\text{mm}}}{\left(\text{δx}\right)^{2}}\left(V_{m}\left[i\right]\left[j-1\right]-V_{m}\left[i\right]\left[j\right]\right)$ | $\frac{G_{\text{gap}}C_{f}}{C_{m}}\left(V_{f}\left[i\right]\left[j-1\right]-V_{m}\left[i\right]\left[j\right]\right)$ |
| $a_{4}$ | $\frac{D_{\text{mm}}}{\left(\text{δx}\right)^{2}}\left(V_{m}\left[i\right]\left[\text{j+1}\right]-V_{m}\left[i\right]\left[j\right]\right)$ | $\frac{G_{\text{gap}}C_{f}}{C_{m}}\left(V_{f}\left[i\right]\left[\text{j+1}\right]-V_{m}\left[i\right]\left[j\right]\right)$ |

Similarly, when the central cell was an MFB, the diffusion term for Eq.33 was computed with $a_{n}$as listed in Table II.

Table II: Computing the diffusion term for Eq.33

| $a_{n}$ | neighbor: MFB | neighbor: NRVM |
| --- | --- | --- |
| $a_{1}$ | $\frac{D_{\text{ff}}}{\left(\text{δx}\right)^{2}}\left(V_{f}\left[i-1\right]\left[j\right]-V_{f}\left[i\right]\left[j\right]\right)$ | $G_{\text{gap}}\left(V_{m}\left[i-1\right]\left[j\right]-V_{f}\left[i\right]\left[j\right]\right)$ |
| $a_{2}$ | $\frac{D_{\text{ff}}}{\left(\text{δx}\right)^{2}}\left(V_{f}\left[\text{i+1}\right]\left[j\right]-V_{f}\left[i\right]\left[j\right]\right)$ | $G_{\text{gap}}\left(V_{m}\left[\text{i+1}\right]\left[j\right]-V_{f}\left[i\right]\left[j\right]\right)$ |
| $a_{3}$ | $\frac{D_{\text{ff}}}{\left(\text{δx}\right)^{2}}\left(V_{f}\left[i\right]\left[j-1\right]-V_{f}\left[i\right]\left[j\right]\right)$ | $G_{\text{gap}}\left(V_{m}\left[i\right]\left[j-1\right]-V_{f}\left[i\right]\left[j\right]\right)$ |
| $a_{4}$ | $\frac{D_{\text{ff}}}{\left(\text{δx}\right)^{2}}\left(V_{f}\left[i\right]\left[\text{j+1}\right]-V_{f}\left[i\right]\left[j\right]\right)$ | $G_{\text{gap}}\left(V_{m}\left[i\right]\left[\text{j+1}\right]-V_{f}\left[i\right]\left[j\right]\right)$ |

**Modeling cellular variability:**

To incorporate cellular variability, the following protocol was used: In our model, there are 10 ionic conductances, corresponding to the 10 major and minor ionic currents. A random number generator was used to generate 10 random numbers in the range 0.5-1.5, for each grid point location. Thus, if *N* be the number of grid points outside the circle of interest, $10\times\left(576\times576-N\right)$random numbers were generated in the given range. At each grid point of the simulation domain, within the circle of interest, the 10 ionic conductances were multiplied with 10 of these random numbers. This heterogeneous configuration of the system was saved and used as initial condition for the actual simulations to follow. Thus in our simulations, the electrophysiological properties at every grid point location, were made different from each other, as in the in vitro situation, where cells display a natural heterogeneity in their ionic properties.

**Experimental Methods**

**Animal Studies**

All animal experiments were approved by the Animal Experiments Committee of Leiden University Medical Center and conformed to the Guide for the Care and Use of Laboratory Animals as stated by the United States National Institutes of Health.

**Cell Isolation and Culture**

Neonatal rat ventricular cardiomyocytes (NRVMs) were isolated as previously described.^3^ 2-Day old neonatal Wistar rat pups were anesthetized by inhalation of 5% isoflurane gas, after which adequate anesthesia was confirmed by the absence of reflexes. Hearts were rapidly excised, atria and annuli fibrosi cordis were removed, and the remaining ventricles were finely minced and dissociated with collagenase type 1 (450 U/mL; Worthington, Lakewood, NJ) and DNase I (18.75 Kunitz/mL; Sigma-Aldrich, St. Louis, MO). The cell suspension was transferred to Primaria cell culture dishes (Becton Dickinson, Breda, the Netherlands), which were incubated for 75 minutes at 37ºC and 5% CO_2_ to allow for preferential attachment of non-myocytes (predominantly cardiac fibroblasts [CFBs]). Unattached cardiac cells (mainly NRVMs) were collected and passed through a cell strainer (70-µm mesh pore size; BD Biosciences, Breda, the Netherlands) to obtain a single cell suspension. The attached cells (mainly CFBs) were cultured for one week before establishing co-cultures of these cells, which by this time had obtained characteristics of MFBs,^4^ with freshly isolated NRVMs (*i.e.*, NRVMs from a subsequent round of isolation). To establish co-cultures, the MFBs were dissociated with 0.05% trypsin/EDTA (Life Technologies Europe, Bleiswijk, the Netherlands) and subsequently passed through a 70-µm mesh pore size cell strainer. Single cell counting for both NRVM and MFB suspensions was performed using a BD Accuri C6 flow cytometer (BD Biosciences). NRVM and MF suspensions were mixed in ratios to obtain a 70% NRVMs and 30% MFBs and the resulting mixtures were seeded in 24-well cell culture plates (Corning Life Sciences, Amsterdam, the Netherlands) onto bovine fibronectin (Sigma-Aldrich)-coated round glass coverslips (15 mm diameter). Plating density was 1-7×10^5^ cells/well, depending on the assay. After 24 hours, cells were incubated with mitomycin-C (10 µg/mL; Sigma-Aldrich) for 2 hours to inhibit cell proliferation, as described previously.^4^ Culture medium consisted of Dulbecco’s modified Eagle’s medium (DMEM)/Ham’s F10 medium (1:1, v/v; Life Technologies Europe) supplemented with 5% horse serum (Life Technologies Europe), 2% bovine serum albumin (BSA) and sodium ascorbate to a final concentration of 0.4 mM and was refreshed daily.

**Construction of Self-Inactivating Lentiviral Vector (SIN-LV) Shuttle Plasmids**

Selective knock-down of rat connexin43 (Cx43) expression was accomplished by RNA interference using SIN-LVs encoding rat Gja1 (rGja1) gene-specific short hairpin (sh) RNAs. To this end, the 1.9-kb SphI×EcoRI fragments of SIN-LV shuttle plasmid clones TRCN0000348381 and TRCN0000068474 from the MISSION shRNA library (Sigma-Aldrich) were ligated to the 5.7-kb SphI×EcoRI fragment of pLKO.1-PpLuc-shRNA.hEEF1A1.eGFP^5^ to generate pLKO.1-rGja1-shRNA348381.hEEF1A1.eGFP and pLKO.1-rGja1-shRNA068474.hEEF1A1.eGFP, respectively. pLKO.1-PpLuc-shRNA.hEEF1A1.eGFP, pLKO.1-rGja1-shRNA348381.hEEF1A1.eGFP and pLKO.1-rGja1-shRNA068474.hEEF1A1.eGFP were subsequently used to generate particles of the SIN-LVs LV-Ppluc↓, LV-Cx43↓ (ocassionally also named LV.Cx43_1_↓) and LV-Cx43_2_↓, respectively. The correctness of the SIN-LV shuttle plasmids was verified by restriction mapping using 5 different enzymes and by partial nucleotide sequence analysis using the Quick Shot sequencing services of BaseClear (Leiden, the Netherlands) and the human *RNU6-1* gene-specific primer 5’ GACTATCATATGCTTACCGT 3’. Restriction endonucleases and other DNA modifying enzymes were obtained from Thermo Fisher Scientific (Landsmeer, the Netherlands) or New England Biolabs (Bioké, Leiden, the Netherlands). For large-scale purification of the SIN-LV shuttle and packaging plasmids the JETSTAR 2.0 Plasmid Maxiprep kit (Genomed, Löhne, Germany) was used following the instructions of the manufacturer.

**SIN-LV Production and Transduction**

LV-Cx43_1_↓, LV-Cx43_2_↓ and LV-PpLuc↓ particles were produced in 293T cells from SIN-LV shuttle plasmids pLKO.1-rGja1-shRNA348381.hEEF1A1.eGFP, pLKO.1-rGja1-shRNA068474.hEEF1A1.eGFP and pLKO.1-PpLuc-shRNA.hEEF1A1.eGFP, respectively, as described previously.^5^ SIN-LV particles were concentrated by ultracentrifugation and subsequently suspended in phosphate-buffered saline (PBS) containing 1% BSA fraction V (Sigma-Aldrich). SIN-LV suspensions were stored in 100 µL portions at -80ºC until use. Four days after culture initiation, cells were transduced by adding different volumes of SIN-LV suspension directly to culture medium. At 24 hours after vector addition, the inoculum was removed, the monolayers were washed 3 times with culture medium and kept in fresh culture medium until structural or functional analysis at day 9 of culture. Transduction efficiency was determined by assessment of enhanced green fluorescent protein (eGFP) fluorescence with an Axiovert 200M inverse fluorescence microscope (Carl Zeiss, Sliedrecht, the Netherlands). SIN-LVs were applied in a dose range that resulted in transduction of nearly 100% of cells at the lowest vector dose and did not cause microscopic signs of cytotoxicity at the highest vector dose.

**Immunocytology**

Cells were washed with PBS before fixing with PBS/4% formaldehyde (Merck, Amsterdam, the Netherlands) for 15 minutes at room temperature (RT) followed by 3 washes with PBS. For transduction efficiency assessment, cells immediately underwent nuclear counterstaining by incubation with 10 µg/mL Hoechst 33342 (Life Technologies Europe) for 10 minutes at RT. Coverslips were mounted in Vectashield mounting medium (Vector Laboratories, Burlingame, CA). For all other experiments, cells were permeabilized by incubation with PBS/0.05% Triton-X100 (Sigma-Aldrich) for 10 minutes at RT. After 3 washes with PBS/0.1% Tween-20, samples were incubated with primary antibodies diluted in PBS/1% donkey serum (DS)/1% BSA. Antibodies against the following antigens were used: sarcomeric α-actinin (Actn2 gene product) to detect NRVMs (1:200; mouse IgG1, clone EA-53; Sigma-Aldrich), collagen type I (Col1a1 gene product) to identify fibroblastic cell types (1:200; rabbit IgG, polyclonal; Abcam, Cambridge, United Kingdom), CD31 (Pecam1 gene product) to identify endothelial cells (1:200; rat IgG2a, clone MEC 13.3; BD Biosciences), smooth muscle myosin heavy chain (Myh11 gene product) to detect smooth muscle cells (1:200; mouse IgG1, clone hSM-V; Sigma-Aldrich), and the gap junctional protein Cx43 (Gja1 gene product;1:200; rabbit IgG, polyclonal; Sigma-Aldrich). After washing 3 times for 5 minutes with PBS/0.1% Tween-20, cells were incubated with appropriate Alexa Fluor 488/568-conjugated secondary antibodies (1:500; Life Technologies Europe) or, for detecting CD31, with biotinylated rabbit-anti rat IgG secondary antibodies (1:200; Vector Laboratories) and Alexa Fluor 488 streptavidin conjugates (1:200; Life Technologies). Nuclear counterstaining and mounting of the coverslips in Vectashield were performed as described before. Images were acquired with a Leica TCS SP8 confocal laser scanning microscope (Leica, Solms, Germany). Storage and quantification of immunofluorescence signals was done using dedicated software (Leica Application Suite [Leica], NIS Elements [Nikon Instruments Europe] and Fiji [www.fiji.sc]). Each experiment was performed on at least 3 independent cultures.

**Reverse Transcription-Quantitative Polymerase Chain Reaction (RT-qPCR) Analysis**

Dedicated cell cultures were used for RT-qPCR experiments, which were performed essentially as described previously.^6^ In brief, cells were lysed using TRIzol reagent (Life Technologies Europe) and total RNA was isolated with the RNeasy Mini kit (Qiagen, Venlo, the Netherlands). Reverse transcription was performed with the iScript cDNA synthesis kit (Bio-Rad Laboratories, Veenendaal, the Netherlands). cDNA amplification was carried out with the Bioline SensiFAST SYBR No-ROX kit (GC biotech, Alphen aan den Rijn, the Netherlands), using the following oligonucleotides: 5’ GGGATAAGGGAGGTACACA 3’ (rGja1 forward primer) and 5’ CACTCAATTCATGTACACAGACT 3’ (rGja1 reverse primer). For normalization purposes, rat 18S rRNA (Rn18s)-specific cDNA was amplified in parallel using the following primers: 5’ GTAACCCGTTGAACCCCATT 3’ (18S rRNA forward primer) and 5’ CCATCCAATCGGTAGTAGCG 3’ (18S rRNA reverse primer). PCR amplifications were performed using a CFX96 Touch Real-Time PCR detection system (Bio-Rad Laboratories). For data analysis, dedicated software was used for data storage and analysis (CFX Manager Software version 3.1 [Bio-Rad Laboratories]).

**Western Blotting**

Western blotting was performed as previously described.^7^ Cells were washed 3 times with ice-cold PBS and lysed in 150 mM NaCl, 1% Triton X-100, 0.5% sodium deoxycholate, 0.1% sodium dodecyl sulfate, 50 mM Tris-HCl (pH 8.0) supplemented with protease inhibitors (cOmplete, Mini Protease Inhibitor Cocktail Tablet; Roche Applied Science, Penzberg, Germany). After centrifugation for 15 minutes at 21,130×*g* and 4ºC, the supernatant was collected, passed 3 times through a sterile syringe with 29G needle (BD Biosciences), aliquoted and stored at -80ºC until assay. Protein concentration was determined using the BCA protein assay kit (Thermo Fisher Scientific). Proteins were size-fractionated in Novex Bolt 8% Bis-Tris Plus gels (Life Technologies Europe) and transferred to Amersham Hybond-N^+^ polyvinylidene difluoride membranes (GE Healthcare, Diegem, Belgium) by wet electroblotting using a Bolt Mini blot module (Life Technologies Europe). After blocking for 1 hour in 2% ECL Prime blocking agent (GE healthcare) dissolved in Tris-based saline/0.1% Tween-20 (TBST), membranes were incubated for 1 hour with primary antibodies directed against Cx43 (1:100,000; rabbit IgG, polyclonal; Sigma-Aldrich) or lamin A/C (Lmna gene product; 1:5,000; rabbit IgG, polyclonal; Santa Cruz Biotechnology, Dallas, TX) as an internal control. All antibodies were diluted in TBST/2% ECL Prime blocking agent. After 3 times washing with TBST, blots were incubated with corresponding horseradish peroxidase-conjugated secondary antibodies (1:25,000; donkey-anti-rabbit IgG-HRP; Santa Cruz Biotechnology) for 1 hour at RT. After 3 wash steps with TBST, membranes were immersed in SuperSignal West Femto maximum sensitivity substrate (Thermo Fisher Scientific). Chemiluminescence was measured with the ChemiDoc Touch imaging system (Bio-Rad Laboratories), whose software was used for data storage and quantificative analysis.

**Optical Voltage Mapping**

Assessment of the effects of Cx43 knockdown on cardiac electrical impulse generation and propagation in monolayers was done by optical mapping on day 9 of culture. Cells were loaded with the potentiometric dye di-4-ANEPPS (8 µM final dye concentration; Life Technologies) in serum-free and phenol red-less DMEM/Ham’s F12 medium (1:1, v/v; Life Technologies) by incubation for 15 minutes at 37ºC in a humidified incubator (95% air/5% CO_2_). Optical mapping experiments were performed in fresh unsupplemented DMEM/Ham’s F12 medium at 37ºC, using a MiCAM ULTIMA-L imaging system (SciMedia, Costa Mesa, CA). Cells were stimulated electrically with an epoxy-coated bipolar platinum electrode with square 10- ms, 8-V suprathreshold electrical stimuli using a STG 2004 stimulus generator and MC Stimulus II software (both from Multichannel Systems, Reutlingen, Germany). Optical signals were recorded at a 6-ms frame rate and analyzed using BrainVision Analyzer 13.12.20 software (Brainvision, Tokyo, Japan), after spatial and temporal filtering. Cultures were stimulated at pacing frequencies of 0.5, 1, 2, 3, 4 and 5 Hz, and assessed for CV, CV dispersion, action potential duration (APD) at 80% of full repolarization (APD_80_) and APD_80_ dispersion of repolarization. Fibrillation was induced by burst pacing at cycle lengths of 70-350 ms. Wavelength was calculated by multiplying average CV with APD_80_ (for paced propagation) or with reentrant cycle length. Complexity was determined by quantifying phase singularities (PSs) per culture. Phase maps were constructed with dedicated software using the phase space method, as described previously.^8^

**Statistical Analysis**

Statistical analyses were performed using GraphPad Prism software version 6 (Graphpad Software, La Jolla, CA). Unpaired Student’s t test, Fisher’s exact test and the one-way ANOVA test were used for comparing different experimental groups. Data were expressed as mean±standard error of mean (SEM) for a specified number (N) of observations. Results were considered statistically significant at p values<0.05. Statistical significance was expressed as follows: *: *P*<0.05, **: *P*<0.001 and NS: not significant.

**References**

1. Korhonen, T., Hanninen, S.L. & Tavi, P. Model of excitation-contraction coupling of rat neonatalventricular myocytes. *Biophys J.* **96**, 1189-1209 (2009).
2. Hou, L., *et al*. A major role for HERG in determining frequency of reentry in neonatal rat ventricular myocyte monolayer. *Circ Res.* **107**, 1503-1511 (2010).
3. Askar, S.F., *et al*. Engraftment patterns of human adult mesenchymal stem cells expose electrotonic and paracrine proarrhythmic mechanisms in myocardial cell cultures. *Circ Arrhythm Electrophysiol.* **6**, 380-391 (2013).
4. Askar, S.F., *et al*. Antiproliferative treatment of myofibroblasts prevents arrhythmias in vitro by limiting myofibroblast-induced depolarization. *Cardiovasc Res.* **90**, 295-304 (2011).
5. Bingen, B.O., *et al*. Atrium-specific Kir3.x determines inducibility, dynamics, and termination of fibrillation by regulating restitution-driven alternans. *Circulation.* **128**, 2732-2744 (2013).
6. Engels, M.C., *et al*. Insulin-like growth factor promotes cardiac lineage induction in vitro by selective expansion of early mesoderm. *Stem Cells.* **32**, 1493-1502 (2014).
7. Engels, M.C. *et al.* Forced fusion of human ventricular scar cells with cardiomyocytes suppresses arrhythmogenicity in a co-culture model. *Cardiovasc Res.* **107**, 601-612 (2015).
8. Bingen, B.O., *et al*. Prolongation of minimal action potential duration in sustained fibrillation decreases complexity by transient destabilization. *Cardiovasc Res.* **97**, 161-170 (2013).

**Supplementary Figures**

**Figure S1.** Mathematical model of NRVM. **A**, The connectivity of cells in the mathematical monolayer was modeled as a 5-point stencil, with each cell site being occupied by either a cardiomyocyte or a MFB, but not both. The central cell is labeled with index (i,j); neighboring cells are indexed as shown in the figure. **B,** Comparison of the action potential morphology and (**C**) action potential characteristics in the present model and in the model by Hou *et al*.^2^ **D**, APD_80_ restitution curve of present model, compared with the *in silico* (model) and *in vitro* (exp) results obtained by Hou *et al*.
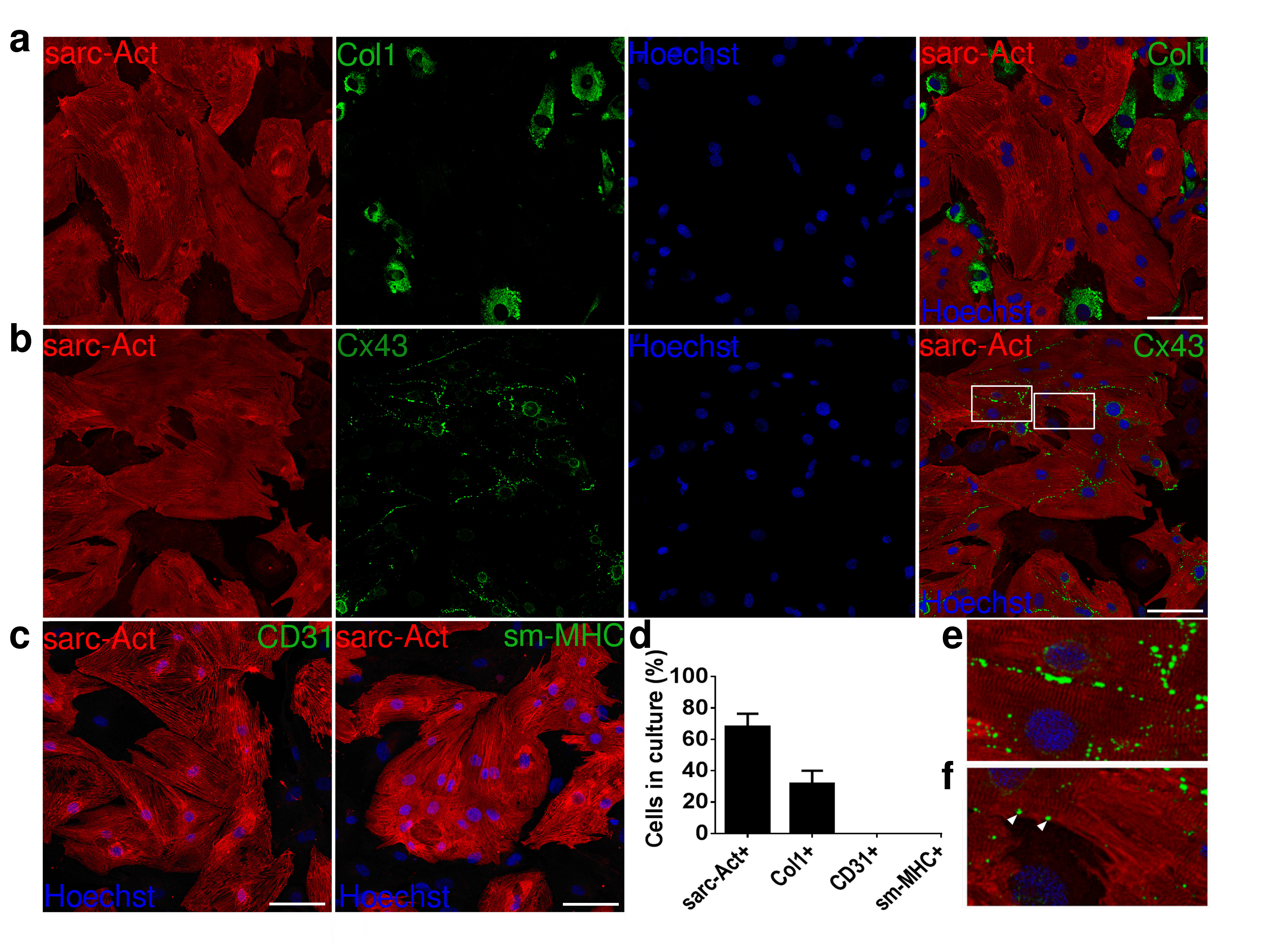
2

**Figure S2.** Immunocytological characterization of the *in vitro* model. **A**, Confocal microscopy images of immunocytological staining for sarcomeric alpha actinin (sarc-Act; red) and collagen type I (Col1; green) combined with Hoechst 33342 (nuclei, blue) show that the co-cultures contain ~70% sarc-Act^+^ cells (*i.e.*, NRVMs) and ~30% fibroblastic cells (*i.e.*, Col1^+^ cells). **B**, Confocal microscopy images of immunocytological staining for sarc-Act (red), Cx43 (green) combined with Hoechst 33342 (nuclei, blue) demonstrate the presence of Cx43 at cell-cell junctions, especially those between NRVMs. **C,** Confocal microscopy images of immunocytological staining for sarc-Act (red), CD31 (left panel; green) or smooth muscle myosin heavy chain (sm-MHC; right panel; green) combined with Hoechst 33342 (nuclei, blue) show the absence of endothelial cells (*i.e.*, CD31^+^ cells) and smooth muscle cells (sm-MHC^+^ cells). **D**, Quantification of immunological data. **E,F** High magnification images of the white boxes in subfigure B, showing Cx43 (**E**) between 2 NRVMs, and (**F**) between an NRVM and a MFB. White scale bars represent 100
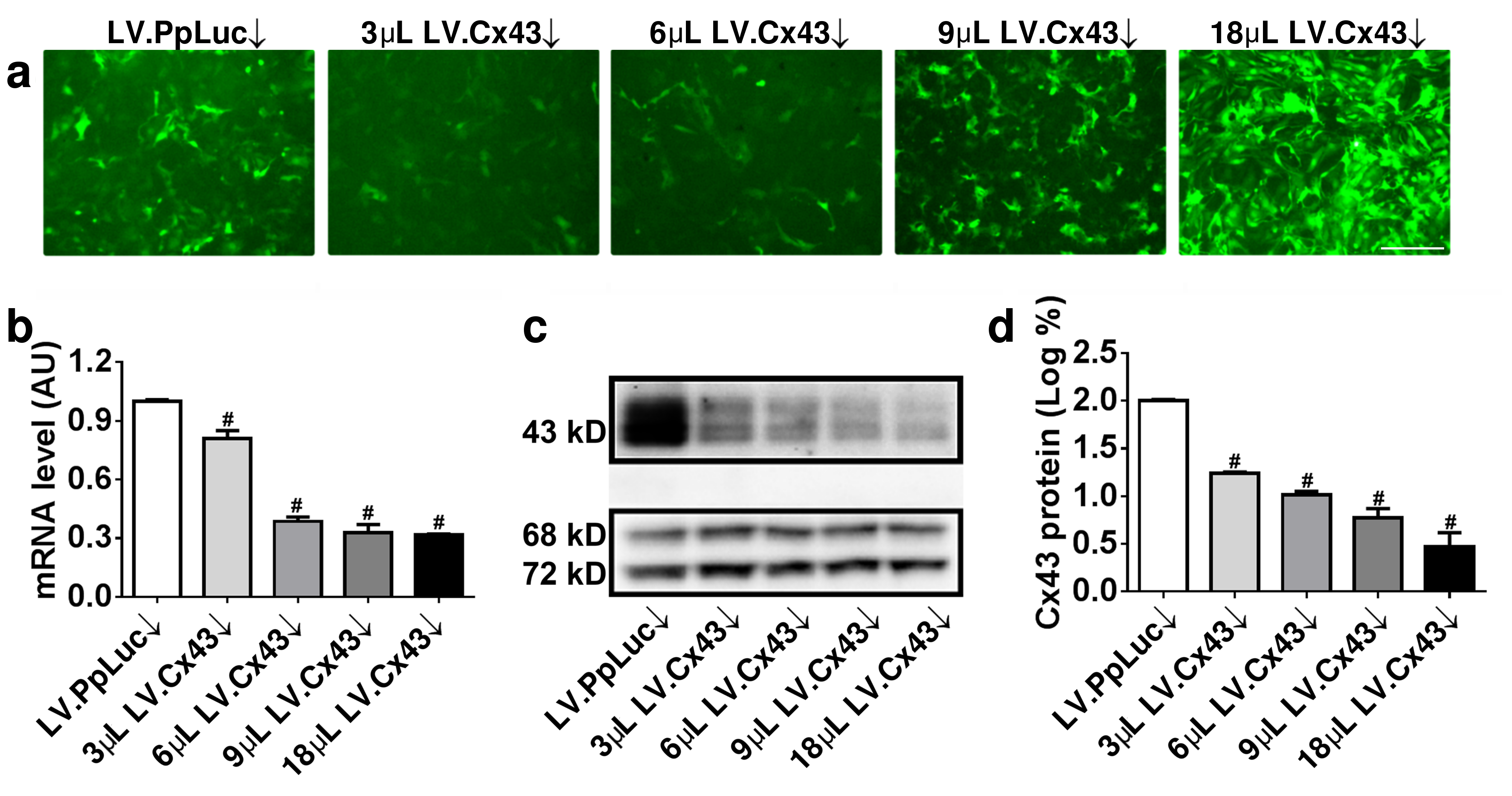
µm.


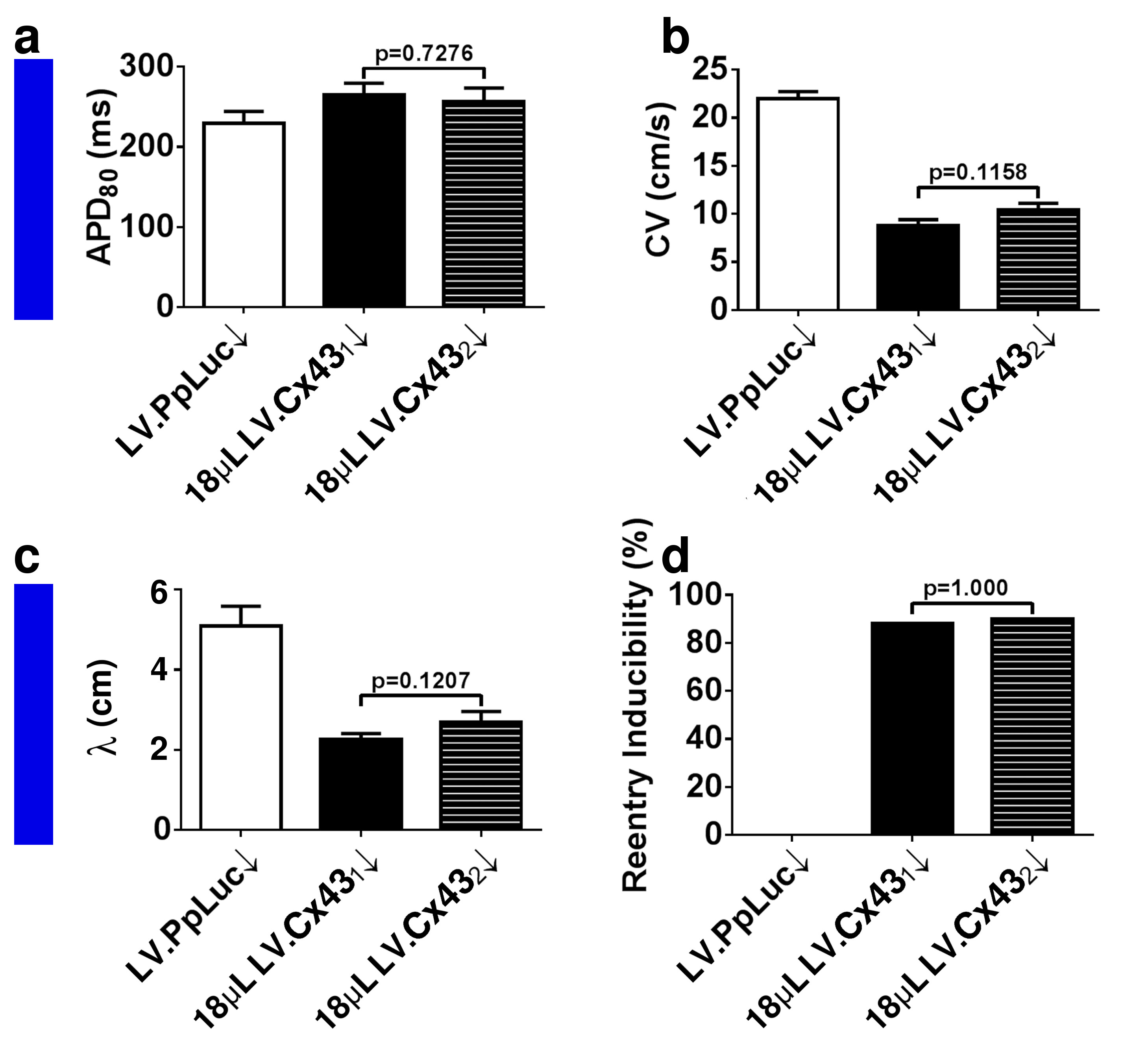


**Figure S3**. Effect of LV.Cx43↓ dose on Cx43 levels. **A**, Fluorographs of NRVM-MFB co-cultures transduced with different doses of LV.Cx43↓ or a single dose of the control vector LV.PpLuc↓. Increasing vector doses result in an increase of eGFP signal intensity. **B**, Relationship between vector dose and rGja1 mRNA level as measured by RT-qPCR in fibrotic NRVM cultures exposed to different amounts of LV.Cx43↓. **C**, Relationship between vector dose and Cx43 protein (43 kDa) level as determined by western blotting using lamin A/C as internal control (68 and 72 kDa protein species). **D**, Quantification of western blot data. #: *P*<0.001. White scale bar represents 100 µm.


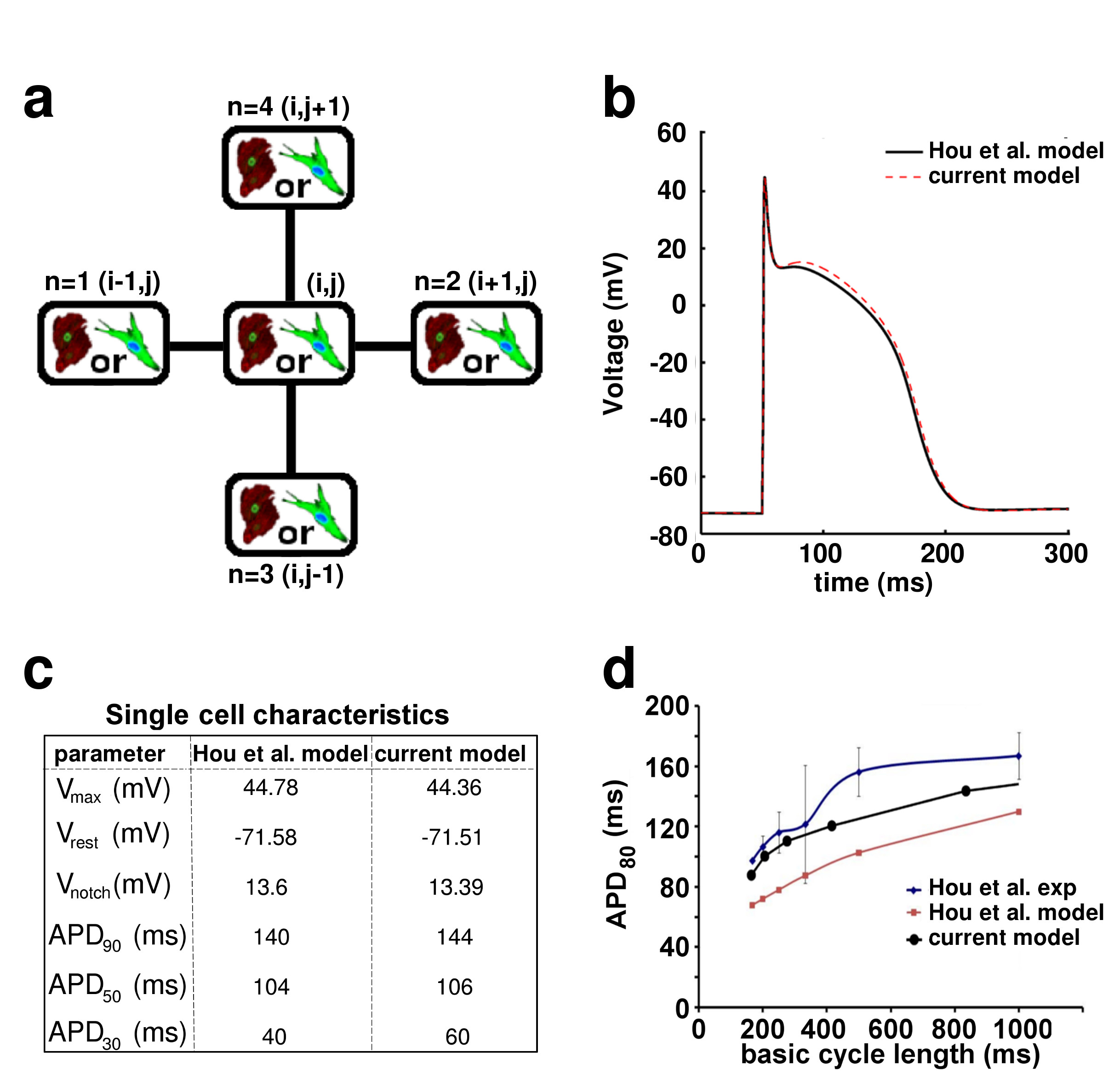


**Figure S4.** Comparison of the electrophysiological effects of SIN-LVs coding for two different rGja1-specific shRNAs. Transduction of fibrotic NRVM cultures with equal volumes of LV.Cx43_1_↓ or LV.Cx43_2_↓ did not result in significant differences in (**A**) APD_80_, (**B**) CV, **(C**) wavelength (λ) and (**D**) reentry inducibility upon rapid pacing. However, CV and λ were considerably smaller and reentry inducibility was much larger than in LV.PpLuc↓-transduced NRVM-MFB co-cultures.
